# Genetic analysis of genes causing hypertension and stroke in spontaneously hypertensive rats: Gene expression profiles in the kidneys

**DOI:** 10.3892/ijmm.2015.2281

**Published:** 2015-07-10

**Authors:** YUKO WATANABE, MOMOKO YOSHIDA, KYOSUKE YAMANISHI, HIDEYUKI YAMAMOTO, DAISUKE OKUZAKI, HIROSHI NOJIMA, TERUO YASUNAGA, HARUKI OKAMURA, HISATO MATSUNAGA, HIROMICHI YAMANISHI

**Affiliations:** 1 Hirakata General Hospital for Developmental Disorders, Hirakata, Osaka 573-0122, Japan; 2 Department of Genome Informatics, Research Institute for Microbial Diseases, Osaka University, Suita, Osaka 565-0871, Japan; 3 DNA-Chip Development Center for Infectious Diseases, Research Institute for Microbial Diseases, Osaka University, Suita, Osaka 565-0871, Japan; 4 Department of Neuropsychiatry, Hyogo College of Medicine, Nishinomiya, Hyogo 663-8501, Japan; 5 Institute for Advanced Medical Sciences, Hyogo College of Medicine, Nishinomiya, Hyogo 663-8501, Japan

**Keywords:** attention-deficit hyperactivity disorder, kidney, gene expression profiles, hypertension, spontaneously hypertensive rats, stroke-prone spontaneously hypertensive rats

## Abstract

Spontaneously hypertensive rats (SHRs) and stroke-prone SHRs (SHRSP) are frequently used as models not only of essential hypertension and stroke, but also of attention-deficit hyperactivity disorder (ADHD). Normotensive Wistar-Kyoto (WKY) rats are normally used as controls in these studies. In the present study, we aimed to identify the genes causing hypertension and stroke, as well as the genes involved in ADHD using these rats. We previously analyzed gene expression profiles in the adrenal glands and brain. Since the kidneys can directly influence the functions of the cardiovascular, endocrine and sympathetic nervous systems, gene expression profiles in the kidneys of the 3 rat strains were examined using genome-wide microarray technology when the rats were 3 and 6 weeks old, a period in which rats are considered to be in a pre-hypertensive state. Gene expression profiles were compared between the SHRs and WKY rats and also between the SHRSP and SHRs. A total of 232 unique genes showing more than a 4-fold increase or less than a 4-fold decrease in expression were isolated as SHR- and SHRSP-specific genes. Candidate genes were then selected using two different web tools: the 1st tool was the Database for Annotation, Visualization and Integrated Discovery (DAVID), which was used to search for significantly enriched genes and categorized them using Gene Ontology (GO) terms, and the 2nd was Ingenuity Pathway Analysis (IPA), which was used to search for interactions among SHR- and also SHRSP-specific genes. The analyses of SHR-specific genes using IPA revealed that B-cell CLL/lymphoma 6 (*Bcl6*) and SRY (sex determining region Y)-box 2 (*Sox2*) were possible candidate genes responsible for causing hypertension in SHRs. Similar analyses of SHRSP-specific genes revealed that angiotensinogen (*Agt*), angiotensin II receptor-associated protein (*Agtrap*) and apolipoprotein H (*Apoh*) were possible candidate genes responsible for triggering strokes. Since our results revealed that SHRSP-specific genes isolated from the kidneys of rats at 6 weeks of age, included 6 genes related to Huntington's disease, we discussed the genetic association between ADHD and Huntington's disease.

## Introduction

Studies have been conducted in an attempt to identify the genes causing hypertension using 2 strains of hypertensive rats: spontaneously hypertensive rats (SHRs) and a substrain derived from the SHRs, stroke-prone SHRs (SHRSP) (1,2). Normotensive Wistar-Kyoto (WKY) rats are normally used as controls in these studies (1). Since SHRs and SHRSP are not only used as models of essential hypertension and stroke, but also as models of attention-deficit hyperactivity disorder (ADHD), it is expected that using these rats, it is possible identify the genes related not only to hypertension and stroke, but also to ADHD (3).

In our previous studies, we investigated gene expression profiles in the adrenal glands (4), and subsequently in the brain (5). Since the kidneys are logical candidate organs for studying hypertension due to their direct influence on body fluids and on the functions of the endocrine, cardiovascular and sympathetic nervous systems, in the present study, we aimed to investigate gene expression profiles in the kidneys. Since the association between kidney function and blood pressure is known to be influenced by numerous intrinsic and extrinsic factors, such as the renin-angiotensin system and catecholamine and aldosterone hormones (6), we compared gene expression profiles in the kidneys of SHRs and WKY rats and also between SHRSP and SHRs, when the rats were at 3 and 6 weeks old, a period in which rats are considered to be in a pre-hypertensive state. We isolated a total of 232 unique genes showing more than a 4-fold increase or less than a 4-fold decrease in expression.

After classifying these 232 genes into 4 groups according to their expression profiles, candidate genes were selected as significantly enriched genes, and categorized with Gene Ontology (GO) terms using the Database for Annotation, Visualization and Integrated Discovery (DAVID) web tools (7,8). Candidate genes were also selected using Ingenuity Pathway Analysis (IPA). The IPA path explorer tool revealed that B-cell CLL/lymphoma 6 (*Bcl6*) (9–13) and SRY (sex determining region Y)-box 2 (*Sox2*) (14,15) were possible candidate genes that trigger hypertension in SHRs. Moreover, our findings revealed that angiotensinogen (*Agt*), angiotensin II receptor-associated protein (*Agtrap*) (16–18) and apolipoprotein H (*Apoh*) (19) played pivotal roles among SHRSP-specific genes.

## Materials and methods

### Animals, RNA extraction, microarray design, microarray analysis and microarray data analysis, reverse transcription-quantitative polymerase chain reaction (RT-qPCR), DAVID and IPA

The details of these procedures have been described in our previous studies [Yamamoto *et al* (4) and Yoshida *et al* (5)].

### Animals

Three strains of rat, SHR/Izm, SHRSP/Izm and WKY/Izm, were provided by the Disease Model Cooperative Research Association, Kyoto, Japan. Three-week-old rats were purchased and maintained for 2 days in our animal facility and were used as 3-week-old rats. Five-week-old rats were purchased and, after being maintained for 1 week in our animal facility, were used as 6-week-old rats.

### RNA extraction

Briefly, total RNA was purified using a miRNeasy kit (Qiagen, Hilden, Germany) according to the manufacturer's instructions.

### Microarray design

Expression profiling was generated using a 4x44K whole rat genome oligo microarray version 3.0 G2519F (Agilent Technologies Inc., Santa Clara, CA, USA). Eighteen microarray analyses as 1 color experiment were performed using the WKY rats, SHRs, and SHRSP at 3 and 6 weeks old as biological triplicates. Each gene expression profile was compared between the SHRs and WKY rats and also between the SHRSP and SHRs.

### Microarray analysis

Total RNA (200 ng) was reverse transcribed into double-stranded cDNA by the AffinityScript Multiple Temperature Reverse Transcriptase (Agilent Technologies Inc.) and amplified. The resulting cDNA was used for *in vitro* transcription by T7-polymerase and labeled with cyanine-3-labeled cytosine triphosphate (Perkin-Elmer, Wellesley, MA, USA) using a Low Input Quick Amp Labeling kit (Agilent Technologies Inc.). After being labeled and fragmented, each cRNA sample was hybridized on Agilent 4×44K whole rat genome arrays (Agilent Design #028282). After washing, the slides were scanned using an Agilent Microarray Scanner (G2505C; Agilent Technologies Inc.). Feature Extraction software (version 10.5.1.1) was used to convert the images into gene expression data.

### Microarray data analysis

Raw data were imported into Subio Platform version 1.12 (Subio Inc., Kagoshima, Japan) and raw intensity data were normalized to the 75th percentile intensity of probes above background levels (gIsWellAbove=1). SHR- and SHRSP-specific genes were defined as those with signal ratios with more than a 4.0-fold increase or less than a 4.0-fold decrease in expression. Raw data have been accepted in the Gene Expression Omnibus (GEO, accession no. GSE41453).

### RT-qPCR

To validate the results obtained by the microarray analysis, 11 enriched genes were randomly selected from 39 enriched unique genes, and RT-qPCR was performed under 15 different experimental conditions. Statistical comparisons between the microarray and RT-qPCR data were performed using Spearman's rank correlation test.

### DAVID web tool analysis

Annotation enrichment analysis was performed using the DAVID (http://david.abcc.ncifcrf.gov/) web tool (version 6.7, 2010) (7,8) with GenBank IDs bearing Entrez Gene ID (Table I, unique genes identified). This web-based resource provides a set of functional annotation tools for the statistical enrichment of genes categorized into GO terms. We used the GO FAT category, which filtered out very broad GO terms to identify statistically enriched functional groups. The annotated gene and protein symbols were written in italic and regular fonts, respectively.

### IPA

IPA software (IPA^®^; Qiagen Redwood City, CA, USA, http://www.qiagen.com/ingenuity) was applied to microarray analyses that were conducted to provide functionality for the interpretation of the gene expression data. IPA was performed with Agilent probe IDs bearing Entrez Gene ID as an input for data (Table I, mapped probes). This web tool was used to overlay functions and diseases, and to categorize SHR- and SHRSP-specific genes according to disease-related or functional annotations. It identified the biological functions and/or diseases in the Ingenuity Knowledge Base (Spring 2014 version) that were the most significant to each of the category sets. The probability of the assignment was expressed by a P-value calculated using the right-tailed Fisher's exact test. The path explorer tool was also used to identify relevant interactions among SHR- and SHRSP-specific genes and to identify the shortest literature-supported paths between genes.

IPA was performed using the IPA database (Spring 2014 release of IPA) and the probe IDs of each gene. The data obtained with DAVID were based on the database (version 6.7, 2010) and GenBank IDs of each gene. Since the renewal dates of these two databases were different, small differences were observed between these two annotation results.

## Results

### Isolation and classification of SHR- and SHRSP-specific genes

We compared gene expression profiles between the SHRs and WKY rats and also between the SHRSP and SHRs, at 3 and 6 weeks of age, and isolated SHR- and SHRSP-specific genes using genome-wide microarray technology. Since we expected the expression of candidate genes to be regulated before elevations in blood pressure (BP), i.e., in the pre-hypertensive period, we examined the expression profiles of each probe using RNA samples prepared from the kidneys, and isolated a total of 353 SHR- and SHRSP-specific probes showing more than a 4-fold increase or less than a 4-fold decrease in expression (Table I).

**Figure 1:**
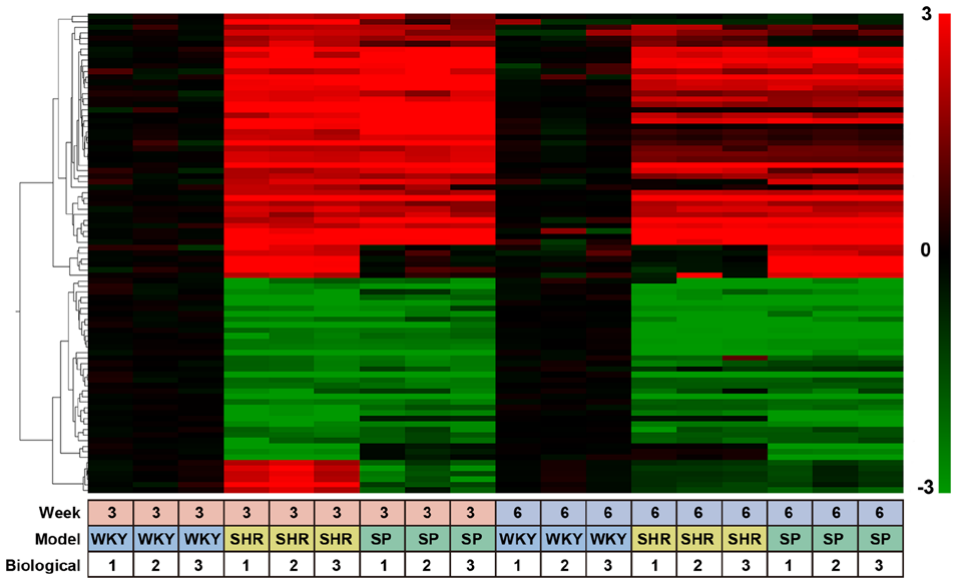
Heatmap of SHR- and SHRSP-specific probes. A heat map of SHR- and SHRSP-specific probes isolated from the kidneys of 3- and 6-week-old rats. Data were obtained with 353 probes for 3 rat strains, WKY rats, SHRs and SHRSP, under 18 different experimental conditions (3 different rat strains, 2 different rat ages, and triplicate experiments). The data obtained with G-1 probes, i.e., 87 out of 353 probes (Table I), were clustered based on their biological function and expression profiles using a hierarchical clustering program and Spearman's rank correlation. The values used for clustering were obtained by microarray experiments as described in the Materials and methods. The color bar at the right side of the panel indicates the log2 ratio for SHRs and SHRSP at 3 or 6 weeks of age vs. WKY rats at 3 or 6 weeks of age. The bottom panel (small boxes) indicates the experimental conditions, i.e., examined at 3 or 6 weeks of age, 3 different rat strains, and triplicate experiments. SHRs, spontaneously hypertensive rats; SHRSP, stroke-prone SHRs; WKY rats, Wistar-Kyoto rats.

We classified the 353 probes into 4 groups, from G-1 to G-4 (Table I). G-1 probes were isolated from the rats at 3 weeks of age and contained 87 SHR-specific probes. Their expression profiles were displayed as a heatmap using the Subio Platform (Fig. 1). These 87 probes corresponded to 69 unique genes, 44 of which showed more than a 4-fold increase and 25 showed less than a 4-fold decrease in expression (Table I). G-2 contained 96 SHR-specific genes isolated from the rats at 6 weeks of age, G-3 contained 35 SHRSP-specific genes isolated from the rats at 3 weeks of age, and G-4 contained 32 SHRSP-specific genes isolated from the rats at 6 weeks of age (Table I).

### Categorization and enrichment of SHR- and SHRSP-specific genes

Using the DAVID web tools, the candidate genes causing hypertension, stroke and ADHD were selected from each group as significantly enriched genes. We isolated a total of 61 enriched genes consisting of 39 unique genes (Table II).

In order to verify the results obtained from microarray analysis, we randomly selected 11 out of the 39 genes (Table III-A), performed 15 real-time RT-qPCR experiments (Table III-B), and compared the results obtained with those of the micro-array experiments by applying Spearman's rank correlation test. The results supported a correlation between the reults of these two different experiments as rs=0.814 with a two-tailed P-value <0.001.

A total of 69 G-1 genes included 26 enriched genes categorized with 3 GO terms: i) GO:0005576 (extracellular region); ii) GO:0008289 (lipid binding); and iii) GO:0055114 (oxidation reduction) (Table II, G-1). A total of 96 G-2 genes included 24 enriched genes categorized with 4 GO terms: i) GO:0003013 (circulatory system process); ii) GO:0055114 (oxidation reduction); iii) GO:0010817 (regulation of hormone levels); and iv) GO:0006775 (fat-soluble vitamin metabolic process) (Table II, G-2).

A total of 35 G-3 genes included 6 enriched genes categorized with 2 GO terms: i) GO:0003013 (circulatory system process); and ii) GO:0051918 (negative regulation of fibrinolysis) (Table II, G-3). A total of 32 G-4 genes included 5 enriched genes categorized with 2 GO terms: i) GO:0051918 (negative regulation of fibrinolysis) and ii) GO:0030097 (hemopoiesis) (Table II, G-4).

Although 26 enriched G-1 genes and 5 G-4 genes did not include genes categorized with circulatory system process, 24 enriched G-2 genes included 7 genes, and 6 enriched G-3 genes included 4 genes categorized with circulatory system process, respectively (Table II).

### Functions and disease-related annotations of SHR- and SHRSP-specific genes

As described above, the SHR- and SHRSP-specific genes were classified into 4 groups (Table I), and then categorized based on disease-related or functional annotations using IPA. The results obtained are summarized in Table IV, and identified among other significantly enriched functional categories, such as ‘endocrine system disorders', ‘cardiovascular disease', ‘cardiovascular system development and function' and ‘hereditary disorder' (Table IV).

G-1 genes included 2 genes, cystic fibrosis transmembrane conductance regulator (*Cftr*) and serine peptidase inhibitor, Kazal type 3 (*Spink3*) categorized as ‘endocrine system disorders (idiopathic pancreatitis)' (Table IV, G-1) (20,21). G-2 genes included 8 genes: angiotensin I converting enzyme (*Ace*), deiodinase, iodothyronine, type II (*Dio2*), acyl-Coenzyme A oxidase 2 (*Acox2*), fin bud initiation factor homolog (*Fibin*), flavin-containing monooxygenase 2 (*Fmo2*), indolethylamine N-methyltransferase (*Inmt*), myosin XVI (*Myo16*) and zinc finger and BTB domain containing 16 (*Zbtb16*) categorized as ‘cardiovascular disease (hypertension)' (Table IV, G-2) (22–24). G-3 genes included 6 genes: *Agt*, *Apoh*, epoxide hydrolase 2 (*Ephx2*), histidine-rich glycoprotein (*Hrg*), ryanodine receptor 1 (*Ryr1*) and vascular endothelial growth factor B (*Vegfb*) categorized as ‘cardiovascular system development and function (development of cardiovascular system)' (Table IV, G-3) (25–30). G-4 genes included 6 genes: Btg3 associated nuclear protein (*Banp*), *Ephx2*, retinoid X receptor gamma (*Rxrg*), *Ryr1*, RNA-binding protein fox-1 homolog 1 (*Rbfox1*) and *Zbtb16* categorized as ‘hereditary disorder (Huntington's disease)' (Table IV, G-4) (31–33).

### Interactions among SHR-specific G-1 and G-2 genes

**Figure 2:**
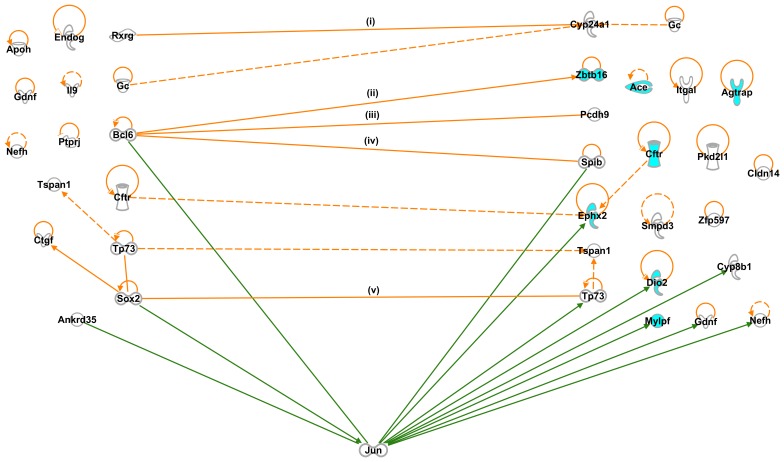
Analyses of interactions among SHR-specific genes. Interactions among SHR-specific genes isolated when the rats were 3 and 6 weeks old were analyzed using IPA. The figure shows the gene-to-gene correlations identified. Genes were represented as nodes and the biological relationship between 2 nodes was represented as an edge (line). Edges signified different correlation's: solid lines represent direct interactions or associations and dotted lines represent indirect interactions or associations. Nodes were displayed using various shapes that represented the functional class of the gene product (refer to IPA for detailed node information). The 15 nodes on the left represented SHR-specific genes isolated at 3 weeks of age and the 21 nodes on the right represented SHR-specific genes isolated at 6 weeks of age. Interactions among SHR-specific genes were represented as orange edges and direct interactions between G-1 and G-2 genes were indicated by (i–v). Interactions after proposing the presence of Jun were represented as green edges. Nodes colored light blue represented BP-regulating genes categorized into GO:0003013 (circulatory system process) by DAVID (Table II) and/or categorized into cardiovascular disease (hypertension) by IPA (Table IV) at 6 weeks of age. SHRs, spontaneously hypertensive rats; SHRSP, stroke-prone SHRs; IPA, Ingenuity Pathway Analysis; BP, blood pressure.

Since our working hypothesis is that G-1 genes include genes that regulate the expression of G-2 genes, we examined the interactions between 69 G-1 and 96 G-2 genes using IPA, and found 5 direct and 3 indirect interactions (Table I and Fig. 2): *Rxrg* and group-specific component (*Gc*) interacted with cytochrome P450 subfamily 24 (*Cyp24a1*) (34,35); *Bcl6* interacted with the following 3 genes: *Zbtb16* (9,10), protocadherin 9 (*Pcdh9*) (11) and Spi-B transcription factor (*Spib*) (12); *Cftr* interacted with *Ephx2* (36); tumor protein p73 (Tp73) interacted with tetraspanin 1 (*Tspan1*) (37); and *Sox2* interacted with *Tp73* (14).

Other than the 8 interactions between the G-1 and G-2 genes, we identified 3 interactions among the G-1 genes: *Tp73* interacted with *Tspan1*; *Sox2* interacted with *Tp73*; *Sox2* interacted with connective tissue growth factor (*Ctgf*) (38); and among the G-2 genes: *Gc* interacted with *Cyp24a1*; *Cftr* interacted with *Ephx2*; *Tp73* interacted with *Tspan1*, respectively. We also found 12 and 16 self-control genes among the SHR-specific G-1 and G-2 genes, respectively (Fig. 2).

However, we did not detect any interactions between the G-1 genes and the majority of BP-controlling G-2 genes, such as *Ace*, *Agtrap*, *Cftr*, glucagon-like peptide 1 receptor (*Glp1r*), kininogen 2 (*Kng2*), myosin light chain, phosphorylatable, fast skeletal muscle (*Mylpf*), *Acox2*, *Dio2*, *Fibin*, *Fmo2*, *Inmt* and *Myo16* (Table II, G-2; GO:0003013, circulatory system process and Table IV, G-2; cardiovascular disease: hypertension).

### Interactions among SHRSP-specific G-3 and G-4 genes

**Figure 3:**
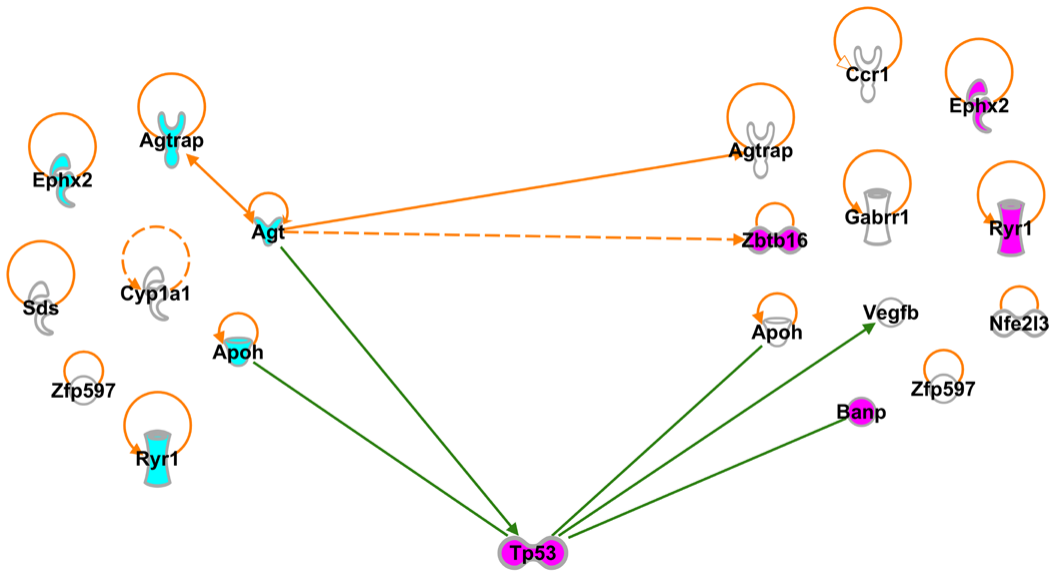
Analyses of interactions among SHRSP-specific genes. Interactions among SHRSP-specific genes isolated when the rats were 3 and 6 weeks of age were analyzed using IPA. The 8 nodes on the left corresponded to SHRSP-specific genes isolated at 3 weeks of age, while the 11 nodes on the right corresponded to SHRSP-specific genes isolated at 6 weeks of age. Nodes colored light blue represented BP-regulating genes categorized into GO:0003013 (circulatory system process) by DAVID (Table II) and/or categorized into cardiovascular system development and function (development of cardiovascular system) by IPA (Table IV) at 3 weeks of age, and nodes colored pink represented Huntington's disease-related genes categorized into hereditary disorder (Huntington's disease) at 6 weeks of age. SHRs, spontaneously hypertensive rats; SHRSP, stroke-prone SHRs; IPA, Ingenuity Pathway Analysis; BP, blood pressure.

Since the enriched G-3 genes were expected to regulate the expression of the G-4 genes, we examined the interactions between 35 G-3 and 32 G-4 genes using IPA, and found that *Agt* interacted not only with *Agtrap* (16,17) expressed in the rats at 3 and 6 weeks of age, but also indirectly interacted with *Zbtb16* (39) expressed in the rats at 6 weeks of age (Fig. 3). In addiiton, a total of 5 self-control genes, such as *Agtrap*, *Ephx2*, *Apoh*, *Ryr1* and zinc finger protein 597 (*Zfp597*) were found to be expressed in the SHRSP at 3 and 6 weeks of age (Fig. 3).

The description and reference of each gene are summarized in Table V.

## Discussion

### General considerations

The first aim of the present study was to identify candidate genes that triggered hypertension in SHRs, the second was to identify genes related to stroke-prone symptoms, and the third was to identify genes related to ADHD. We compared gene expression profiles between SHRs and WKY rats and also between SHRSP and SHRs at 3 and 6 weeks of age, and isolated a total of 232 unique genes showing more than a 4-fold increase or less than a 4-fold decrease in expression as SHR- or SHRSP-specific genes (Table I). We expected a number of these genes to be related to hypertension, susceptibility to stroke and ADHD.

### Interactions among SHR-specific G-1 and G-2 genes

The IPA path explorer tool suggested the presence of 5 direct interactions between 69 G-1 and 96 G-2 genes (Fig. 2): i) *Rxrg* interacted with *Cyp24a1* (34); *Bcl6* interacted with the following 3 genes: ii) *Zbtb16* (9,10), iii) *Pcdh9* (11) and iv) *Spib* (12); and v) *Sox2* interacted with *Tp73* (14).

- *Rxrg* and *Cyp24a1*: *Rxrg* encodes a member of the retinoid X receptor (Rxr) family of nuclear receptors, which are involved in mediating the antiproliferative effects of retinoic acid. This receptor forms dimers with retinoic acid, thyroid hormone and vitamin D receptors, increasing both DNA binding and transcriptional function on their respective response elements. *Cyp24a1* encodes a member of the cytochrome P450 superfamily of enzymes. Cytochrome P450 proteins are monooxygenases that catalyze a number of reactions involved in drug metabolism and the synthesis of cholesterol, steroids and other lipids. By regulating vitamin D3 levels, this enzyme plays a role in calcium homeostasis and the vitamin D endocrine system.
- *Bcl6* and *Zbtb16*: *Bcl6* encodes a zinc finger transcription factor and contains an N-terminal POZ domain. This protein acts as a sequence-specific repressor of transcription, and has been shown to modulate the transcription of START-dependent IL-4 responses in B cells. This protein can interact with various POZ-containing proteins that function as transcription corepressors. *Zbtb16* is a member of the Kruppel C2H2-type zinc-finger protein family and encodes a zinc finger transcription factor that contains nine Kruppel-type zinc finger domains at the carboxyl terminus. This protein is located in the nucleus, is involved in cell cycle progression, and interacts with a histone deacetylase.
- *Bcl6* and *Pcdh9*: *Pcdh9* encodes a member of the protocadherin family, and of transmembrane proteins containing cadherin domains. These proteins mediate cell adhesion in neural tissues in the presence of calcium. The encoded protein may be involved in signaling at neuronal synaptic junctions.
- *Bcl6* and *Spib*: *Spib* encodes a transcriptional activator that binds to the PU-box (5′-GAGGAA-3′) and acts as a lymphoid-specific enhancer.
- *Sox2* and *Tp73*: *Sox2* encodes a member of the SRY-related HMG-box (SOX) family of transcription factors involved in the regulation of embryonic development and in the determination of cell fate. The product of this gene is required for stem-cell maintenance in the central nervous system, and also regulates gene expression in the stomach. *Tp73* encodes tumor protein p53, which responds to diverse cellular stresses to regulate the target genes that induce cell cycle arrest, apoptosis, senescence, DNA repair, and changes in metabolism. The p53 protein is expressed at low levels in normal cells and at high levels in various transformed cell lines, in which it has been suggested to contribute to transformation and malignancy. p53 is a DNA-binding protein that contains transcription activation, DNA-binding and oligomerization domains. It has been postulated to bind to a p53-binding site and activate the expression of downstream genes that inhibit growth and/or invasion, thereby functioning as a tumor suppressor.

Other than these 5 direct interactions between G-1 and G-2 genes, we identified one direct interaction between G-1 genes; *Sox2* interacted with *Ctgf* (38), which encodes a mitogen that is secreted by vascular endothelial cells. This encoded protein plays a role in chondrocyte proliferation and differentiation, cell adhesion in many cell types, and is related to platelet-derived growth factor. *Ctgf* has been linked to the development and progression of diabetic vascular and renal disease. Low-density lipoproteins (LDL) have previously been shown to induce the expression of *Ctgf* in aortic endothelial cells (40) (Fig. 2).

### SHR-specific G-1 and G-2 genes related to hypertension

Even based on the interactions, described above, we were unable to pinpoint the candidate gene(s) causing hypertension. Although these predicted interactions included the hypertension-related G-2 genes, *Ephx2* and *Zbtb16*, they did not include other hypertension-related genes, such as *Ace*, *Agtrap*, *Cftr*, *Glp1r*, *Kng2*, *Mylpf*, *Acox2*, *Dio2*, *Fibin*, *Fmo2*, *Inmt* and *Myo16* (Table II, G-2; GO:0003013, circulatory system process and Table IV, G-2; cardiovascular disease: hypertension). In order to identify further interactions between SHR-specific G-1 and G-2 genes, we applied the IPA path explorer tool, suggested the presence of one gene that assisted in these interactions, and found such a condition when we proposed the *Jun* proto-oncogene (*Jun*) (41), which interacts directly with specific target DNA sequences to regulate gene expression. The presence of *Jun* has been shown to facilitate interactions between 3 G-1 genes: *Bcl6* (13), *Sox2* (15) and ankyrin repeat domain 35 (*Ankrd35*) (42), and 8 G-2 genes: *Spib* (43), *Ephx2* (44), *Tp73* (45), *Dio2* (46), cytochrome P450, family 8, subfamily b, polypeptide 1 (*Cyp8b1*) (47), *Mylpf* (48), glial cell derived neurotrophic factor (*Gdnf*) (49) and neurofilament, heavy polypeptide (*Nefh*) (50) (Fig. 2).

These findings suggested that *Bcl6* and *Sox2* were the candidate genes responsible for causing hypertension in SHRs.

### Interactions among SHRSP-specific G-3 and G-4 genes

Since the candidate gene(s) found to cause stroke in SHRSP were expected to be included in the G-3 genes, we focused on the interaction between G-3 and G-4 genes (Fig. 3). Our results revealed that G-3 genes included 3 typical blood pressure-related genes, *Ephx2*, *Kng2* and *Agtrap* (Table II, G-3; GO:0003013, circulatory system process). These 3 genes isolated from the SHRSP at 3 weeks of age were not isolated from the SHRs at 3 weeks of age, but were isolated from the SHRs at 6 weeks of age (Table II). These results indicated that the expression of genes related to BP control proceeds more rapidly in SHRSP than in SHRs during their development.

The IPA path explorer tool revealed one interaction among G-3 genes and 8 self-controlling genes (Fig. 3). One of the G-3 genes, *Agt*, interacted with another G-3 gene, *Agtrap*, and *Agt* also interacted with 2 G-4 genes, *Agtrap* and *Zbtb16* (Fig. 3). These results suggest that *Agt*, *Agtrap* and *Zbtb16* play pivotal roles in causing stroke-prone symptoms. Moreover, G-4 genes including 9 self-controlling genes (Fig. 3), and self-control genes, such as *Agtrap*, *Ephx2*, *Apoh*, *Ryr1* and *Zfp597*, were expressed in the 3- and 6-week-old SHRSP.

In order to detect further interactions between SHRSP-specific G-3 and G-4 genes, we applied the IPA path explorer tool, suggested the presence of one gene that assisted these interactions, and found such a condition when we proposed tumor protein p53 (*Tp53*) (51), which interacts directly with specific target DNA sequences to regulate gene expression. The presence of *Tp53* facilitated interactions between 2 G-3 genes, *Agt* (18) and *Apoh* (19), and 3 G-4 genes, *Apoh*, *Vegfb* (52) and *Banp* (51) (Fig. 3).

### SHRSP-specific G-3 and G-4 genes related to stroke

Four enriched G-3 genes were categorized as GO:0003013 (circulatory system process). These genes were expected to participate in blood pressure control and the pathogenesis of stroke. Moreover, 2 enriched G-3 genes, *Apoh* and *Hrg*, were categorized as GO:0051918 (negative regulation of fibrinolysis) (Table II, G-3). Since *Apoh* has been implicated in various physiological pathways, including lipoprotein metabolism, coagulation and the production of antiphospholipid autoantibodies, we hypothesized that it may participate in the genesis of atherosclerosis and stroke. *Hrg* possesses antimicrobial activity, and the incorporation of *Hrg* into fibrin clots facilitates bacterial entrapment and killing and promotes inflammation. Since vascular inflammation is known to trigger atherosclerosis, *Hrg* influences atherosclerosis and susceptibility to strokes.

Two out of the 5 enriched G-4 genes, *Apoh* and *Hrg* were categorized as GO:0051918 (negative regulation of fibrinolysis), while the remaining 3 genes, chemokine (C-C motif) receptor 1 (*Ccr1*), leukocyte immunoglobulin-like receptor B-3-like (*Lilrb3l*) and *Zbtb16* were categorized as GO:0030097 (hemopoiesis) (Table II, G-4): *Ccr1* encodes a member of the β-chemokine receptor family. Knockout studies on the mouse homolog suggested roles for this gene in host protection from inflammatory responses, and susceptibility to viruses and parasites. *Lilrb3l* is a receptor for the major histocompatibility complex class I antigen (MHC-I), and may play a physiological role in the brain for neuronal circuitry stability by inhibiting synaptic plasticity. *Zbtb16* encodes a protein which is located in the nucleus. It is involved in cell cycle progression and interacts with a histone deacetylase.

### Genes related to ADHD and Huntington's disease

We previously examined gene expression profiles in the brain, and found that 6 SHRSP-specific genes isolated from the rats at 6 weeks of age (*Agtr1b*, *Arc*, *Egr2*, *Fos*, *Hspa1b* and *Snca*) were annotated to ‘behavior' and were suggested to participate in the genesis of ADHD (5). In the present study, we investigated gene expression profiles in the kidneys, and unexpectedly found that 6 SHRSP-specific genes isolated from the rats at 6 weeks of age (*Banp*, *Ephx2*, *Rbfox1*, *Rxrg*, *Ryr1* and *Zbtb16*) were annotated to ‘Huntington's disease' (Table IV, G-4). *Tp53* was also found to be involved in ‘Huntington's disease' (33,52). These findings suggested the participation of common genes in the genesis of symptoms related to ADHD and Huntington's disease (Table IV, G-4).

### Conclusion

SHR-specific genes isolated from the kidneys of 3-week-old rats included possible candidate genes that trigger hypertension (*Bcl6* and *Sox2*), and SHRSP-specific genes isolated from the kidneys of 3-week-old rats included possible candidate genes that trigger stroke, such as *Agt*, *Agtrap* and *Apoh*. The results obtained from SHRSP-specific genes isolated from the kidneys of 6-week-old rats included 6 genes that have been functionally annotated to Huntington's disease (*Banp*, *Ephx2*, *Rbfox1*, *Rxrg*, *Ryr1* and *Zbtb16*). These results implicate these genes in the involuntary movement associated with Huntington's disease as well as ‘attention-deficit hyperactivity' observed in ADHD.

## Figures and Tables

**Table I:** Comparison of the number and classification of SHR- and SHRSP-specific probes between the 2 pairs of rat strains.

	SHRs/WKY rats		SHRSP/SHRs		All
	G-1 3 weeks old	G-2 6 weeks old	G-3 3 weeks old	G-4 6 weeks old	
All probes isolated	87	156	57	53	353
Mapped probes	72	102	35	32	241
Unmapped probes	15	54	22	21	112
Unique genes identified	69	96	35	32	232
Upregulated	44	51	18	19	132
Downregulated	25	45	17	13	100
Enriched GO terms	3	4	2	2	11
Enriched genes	26	24	6	5^a^	61

Number of SHR- and SHRSP-specific probes isolated from kidneys as described in the Materials and methods section; 232 out of the 353 isolated probes corresponded to unique genes with Entrez Gene IDs. Using DAVID web tools, 232 unique genes were categorized based on GO terms and 11 significantly enriched GO terms, which included 61 enriched genes, were identified (Table II).

a Three of these 5 genes were categorized into GO:0030097 (hemopoiesis) with P=0.0393 (Table II, G-4). SHRs, spontaneously hypertensive rats; SHRSP, stroke-prone SHRs; GO, Gene Ontology; WKY rats, Wistar-Kyoto rats.

**Table II:** Classification and enrichment of SHR- and SHRSP-specific genes.

Group	GO accession	GO term	P-value	Gene symbol	Genes (n)
G-1	GO:0005576	Extracellular region	2.21E-03	*Apoh, Ctgf, Fibin, Gc, Gdnf, Hsd17b13, LOC360919, Nxph1, Serpina3m, Spink3, Spock2, Ucma, Vegfb*	13
	GO:0008289	Lipid binding	3.41E-03	*Acox2, Apoh, Cyp4a2, Gc, Rxrg, Snap91*	6
	GO:0055114	Oxidation reduction	8.72E-03	*Acox2, Akr1c12l1, Cyp4a2, Dhrs7, Hsd17b13, Oxnad1, Rdh16*	7
G-2	GO:0003013	Circulatory system process	4.50E-05	*Ace, Agtrap, Cftr, Ephx2, Glp1r, Kng2, Mylpf*	7
	GO:0055114	Oxidation reduction	3.81E-03	*Acox2, Cyp24a1, Cyp2c11, Dio2, Fmo2, Hsd17b13, Oxnad1, Rdh16, Rdh7*	9
	GO:0010817	Regulation of hormone levels	4.26E-03	*Ace, Dio2, Glp1r, Rdh16, Smpd3*	5
	GO:0006775	Fat-soluble vitamin metabolic process	1.00E-02	*Cyp24a1, Gc, Rdh16*	3
G-3	GO:0003013	Circulatory system process	1.46E-03	*Agt, Agtrap, Ephx2, Kng2*	4
	GO:0051918	Negative regulation of fibrinolysis	5.94E-03	*Apoh, Hrg*	2
G-4	GO:0051918	Negative regulation of fibrinolysis	5.61E-03	*Apoh, Hrg*	2
	GO:0030097	Hemopoiesis	3.93E-02	*Ccr1, Lilrb3l, Zbtb16*	3

SHR- and SHRSP-specific genes were classified into 4 groups (Table I). The members of each group were further categorized with GO terms using DAVID web tools, and genes with significantly enriched GO terms (P<0.01) were identified (except for G-4, GO:0030097). SHRs, spontaneously hypertensive rats; SHRSP, stroke-prone SHRs; GO, Gene Ontology.

**Table III:** Validation of microarray data with RT-qPCR data.

A, Primers used for RT-qPCR experiments		
Gene symbol	Forward primer (5′→3′)	Reverse primer (5′→3′)
*Acox2*	AGGATGCCATCTTGTTAACCGAT	GCCCACTCAAACAGGCGTTC
*Apoh*	CCGGAATCTTAGAAAATGGAGTTGTACGCTA	ACAAGCAAAGCCAATGGTGT
*Cftr*	AGCACACTGAACATCACCGAAG	CACCGTGGGGATCTTTACCAT
*Cyp4a2*	CTCCAGCCTGCTTACCCAT	ATTCATGATGCCGATTGTCCCA
*Gc*	CAAGAAATGTGTGCAGATTATTCCGAGA	TCCTCAGTCGTTCCGCCAA
*Gdnf*	AGTGACTCCAATATGCCCGAAG	CCGCCGCTTGTTTATCTGGT
*Hsd17b13*	CTGAACGTGTCTTAAAAGCTATAAACCGTA	CACACGTCTCTGCACGCAAG
*LOC360919*	TATTAGAGGAATATCTGCAAGGCATCGTCA	ATCAATTCTACCGCGCTTGCT
*Oxnad1*	ACGTTACAAAACAGACGACCCAA	AGTCTCTGCCGAAATGTGCTC
*Rxrg*	CTGTCCCCAAAATGTGATGCTTG	AGATCTCAGCCCCTTAAGTAGCAA
*Ucma*	ACAACCGCCAAAACATATGACC	TGCTTCTCTTCCCCAGTTGCT

B, Data used for Spearman's correlation test				
Group	GenBank ID	Gene symbol	FC (RT-qPCR)	FC (microarray)
G-1	NM_001009626	*Apoh*	−10.236	−61.982
G-1	NM_001044770	*Cyp4a2*	4.808	12.786
G-1	NM_012564	*Gc*	2.438	7.929
G-1	NM_019139	*Gdnf*	−1.036	−5.658
G-1	NM_001009684	*Hsd17b13*	−2.432	−9.747
G-1	NM_001108356	*LOC360919*	−1.252	−5.247
G-1	NM_031765	*Rxrg*	−3.023	−10.778
G-1	NM_001106121	*Ucma*	2.370	7.026
G-2	NM_145770	*Acox2*	2.908	7.567
G-2	NM_031506	*Cftr*	5.125	6.202
G-2	NM_012564	*Gc*	3.866	7.719
G-2	NM_001009684	*Hsd17b13*	−2.097	−11.823
G-2	NM_001107295	*Oxnad1*	−1.199	23.616
G-3	NM_001009626	*Apoh*	9.843	51.420
G-4	NM_001009626	*Apoh*	−8.046	−69.883

RT-qPCR, reverse transcription-quantitative polymerase chain reaction; FC (RT-qPCR), fold change based on the results obtained with RT-qPCR; FC (microarray), fold change based on the results obtained with microarray analyses.

**Table IV:** SHR- and SHRSP-specific genes classified based on disease-related or functional annotations.

Group	Category	Diseases or functions annotation	P-value	Gene symbol	Genes (n)
G-1	Endocrine system disorders	Idiopathic pancreatitis	3.57E-06	*Cftr, Spink3*	2
	Digestive system development and function	Abnormal morphology of duodenum	5.23E-06	*Cftr, Gdnf, Spink3*	3
	Cell cycle	Arrest in cell cycle progression of pheochromocytoma cell lines	7.44E-05	*Galr2, Tp73*	2
	Cellular growth and proliferation	Cytostasis of bone cancer cell lines	9.91E-05	*Bcl6, Tp73*	2
	Cellular growth and proliferation	Stimulation of connective tissue cells	1.51E-04	*Gdnf, Il9, Sox2*	3
	Lipid metabolism	Abnormal quantity of lipids	2.60E-04	*Cftr, Cyp8b1, Gc, Rdh16*	4
G-2	Vitamin and mineral metabolism	Metabolism of vitamin	8.05E-06	*Cyp24a1, Cyp2c11, Gc, Rdh16, Rdh7*	5
	Lipid metabolism	Abnormal quantity of lipid	4.99E-05	*Cftr, Cyp24a1, Cyp8b1, Gc, Rdh16*	5
	Lipid metabolism	Metabolism of 14,15-epoxyeicosatrienoic acid	8.86E-05	*Cyp2c11, Ephx2*	2
	Reproductive system disease	Asthenozoospermia	1.27E-04	*Cftr, Ros1, Tekt3, Zbtb16*	4
	Cardiovascular disease	Hypertension	7.27E-03	*Ace, Acox2, Dio2, Fibin, Fmo2, Inmt, Myo16, Zbtb16*	8
G-3	Lipid metabolism	Quantity of 12-hydroxyeicosatetraenoic acid	7.98E-07	*Agt, Cyp1a1, Ephx2*	3
	Cellular movement	Infiltration by dendritic cells	1.56E-04	*Agt, Hrg*	2
	Molecular transport	Uptake of norepinephrine	2.67E-04	*Agt, Agtrap*	2
	Cardiovascular system development and function	Development of cardiovascular system	1.46E-03	*Agt, Apoh, Ephx2, Hrg, Ryr1, Vegfb*	6
G-4	Hereditary disorder	Huntington's disease	2.71E-05	*Banp, Ephx2, Rbfox1, Rxrg, Ryr1, Zbtb16*	6
	Skeletal and muscular disorders	Skeletal muscle spasticity	3.70E-04	*Gabrr1, Ryr1*	2
	Carbohydrate metabolism	Binding of 1,2-dioleoylphosphatidylserine	7.70E-04	*Apoh*	1
	Cancer	Hypoxia of malignant tumor	7.70E-04	*Hrg*	1
	Cancer	Uterine serous papillary cancer	1.09E-03	*Micb, Nfe2l3, Zbtb16*	3

IPA was used to identify categories (disease or functional annotations) associated with SHR- and SHRSP-specific genes. P-values were calculated using the right-tailed Fisher's exact test in order to determine the significant enrichment of genes in each functional category. The results obtained were aligned while taking the group number, P-values, and molecule numbers into consideration. GenBank gene symbols for each gene are shown. IPA, Ingenuity Pathway Analysis; SHRs, spontaneously hypertensive rats; SHRSP, stroke-prone SHRs.

**Table V:** List of SHR- and SHRSP-specific genes.

Group	GS	Description	GenBank ID	FC	P-value	(Refs.)
G-1	*Acox2*	Acyl-CoA oxidase 2, branched chain	NM_145770	8.194	1.05E-06	
	*Akr1c12l1*	Aldo-keto reductase family 1, member C12-like 1	NM_001135744	4.772	1.11E-04	
	*Ankrd35*	Ankyrin repeat domain 35	XM_001063190	−4.364	9.02E-03	(42)
	*Apoh*	Apolipoprotein H (β-2-glycoprotein I)	NM_001009626	−61.982	2.15E-04	
	*Bcl6*	B-cell CLL/lymphoma 6	NM_001107084	4.010	4.99E-03	(9–13)
	*Cftr*	Cystic fibrosis transmembrane conductance regulator	NM_031506	6.434	4.16E-03	(20,36)
	*Ctgf*	Connective tissue growth factor	NM_022266	4.746	7.61E-04	(38,40)
	*Cyp4a2*	Cytochrome P450, family 4, subfamily a, polypeptide 2	NM_001044770	12.786	6.75E-04	
	*Cyp8b1*	Cytochrome P450, family 8, subfamily b, polypeptide 1	NM_031241	4.822	6.02E-04	
	*Dhrs7*	Dehydrogenase/reductase (SDR family) member 7	NM_001013098	14.138	1.16E-03	
	*Endog*	Endonuclease G	NM_001034938	−4.151	2.54E-05	
	*Fibin*	Fin bud initiation factor homolog (zebrafish)	NM_001025042	4.089	5.41E-03	
	*Galr2*	Galanin receptor 2	NM_019172	−5.289	7.51E-03	
	*Gc*	Group-specific component	NM_012564	7.929	2.12E-05	(35)
	*Gdnf*	Glial cell derived neurotrophic factor	NM_019139	−5.658	4.27E-04	
	*Hsd17b13*	Hydroxysteroid (17-β) dehydrogenase 13	NM_001009684	−9.747	4.47E-05	
	*Il9*	Interleukin 9	NM_001105747	−4.291	7.71E-04	
	*LOC360919*	Similar to α-fetoprotein	NM_001108356	−5.247	8.04E-06	
	*Nefh*	Neurofilament, heavy polypeptide	NM_012607	4.214	8.78E-04	
	*Nxph1*	Neurexophilin 1	NM_012994	4.709	9.34E-03	
	*Oxnad1*	Oxidoreductase NAD-binding domain containing 1	NM_001107295	27.732	3.78E-05	
	*Ptprj*	Protein tyrosine phosphatase, receptor type, J	NM_017269	−4.285	8.21E-05	
	*Rdh16*	Retinol dehydrogenase 16 (all-trans)	NM_199208	5.014	1.25E-03	
	*Rxrg*	Retinoid X receptor gamma	NM_031765	−10.778	1.92E-04	(34)
	*Serpina3m*	Serine (or cysteine) proteinase inhibitor, clade A, member 3M	XM_001067511	21.327	1.09E-05	
	*Snap91*	Synaptosomal-associated protein 91kDa	NM_031728	4.424	2.23E-04	
	*Sox2*	SRY (sex determining region Y)-box 2	NM_001109181	−7.694	1.17E-05	(14,15,38)
	*Spink3*	Serine peptidase inhibitor, Kazal type 3	NM_012674	4.448	1.33E-03	(21)
	*Spock2*	Sparc/osteonectin, cwcv, and Kazal-like domains proteoglycan (testican) 2	NM_001108533	7.515	5.69E-06	
	*Tp73*	Tumor protein p73	NM_001108696	10.360	2.34E-05	(14,37)
	*Tspan1*	Tetraspanin 1	NM_001004236	−6.728	4.26E-05	(37)
	*Ucma*	Upper zone of growth plate and cartilage matrix associated	NM_001106121	7.026	1.06E-05	
	*Vegfb*	Vascular endothelial growth factor B	NM_053549	−340.226	2.71E-06	
G-2	*Ace*	Angiotensin I converting enzyme	NM_012544	−4.207	1.12E-05	(22)
	*Acox2*	Acyl-CoA oxidase 2, branched chain	NM_145770	7.567	1.56E-06	(24)
	*Agtrap*	Angiotensin II receptor-associated protein	NM_001007654	−23.157	2.68E-06	
	*Cftr*	Cystic fibrosis transmembrane conductance regulator	NM_031506	6.202	1.03E-03	(36)
	*Cldn14*	Claudin 14	NM_001013429	−6.099	7.47E-03	
	*Cyp24a1*	Cytochrome P450, family 24, subfamily a, polypeptide 1	BC100059	4.799	1.60E-04	(34,35)
	*Cyp2c11*	Cytochrome P450, subfamily 2, polypeptide 11	NM_019184	9.295	8.44E-03	
	*Cyp8b1*	Cytochrome P450, family 8, subfamily b, polypeptide 1	NM_031241	38.029	2.17E-04	(47)
	*Dio2*	Deiodinase, iodothyronine, type II	NM_031720	5.290	1.47E-03	(23,46)
	*Ephx2*	Epoxide hydrolase 2, cytoplasmic	NM_022936	−10.816	1.56E-05	(36,44)
	*Fibin*	Fin bud initiation factor homolog (zebrafish)	NM_001025042	6.062	7.32E-05	(24)
	*Fmo2*	Flavin-containing monooxygenase 2	NM_144737	5.407	5.45E-05	(24)
	*Gc*	Group-specific component	NM_012564	7.719	6.18E-03	(35)
	*Gdnf*	Glial cell derived neurotrophic factor	NM_019139	−6.710	2.03E-08	(49)
	*Glp1r*	Glucagon-like peptide 1 receptor	NM_012728	−4.181	1.03E-03	
	*Hsd17b13*	Hydroxysteroid (17-β) dehydrogenase 13	NM_001009684	−11.823	8.23E-07	
	*Inmt*	Indolethylamine N-methyltransferase	NM_001109022	5.467	3.43E-06	(24)
	*Itgal*	Integrin αL	NM_001033998	5.943	6.94E-05	
	*Kng2*	Kininogen 2	NM_012741	8.966	3.27E-03	
	*Mylpf*	Myosin light chain, phosphorylatable, fast skeletal muscle	NM_012605	6.139	7.29E-05	(48)
	*Myo16*	Myosin XVI	NM_138893	−19.184	9.42E-05	(24)
	*Nefh*	Neurofilament, heavy polypeptide	NM_012607	4.107	3.37E-06	(50)
	*Oxnad1*	Oxidoreductase NAD-binding domain containing 1	NM_001107295	23.616	1.91E-06	
	*Pcdh9*	Protocadherin 9	NM_001191688	−6.438	2.96E-03	(11)
	*Pkd2l1*	Polycystic kidney disease 2-like 1	NM_001106352	−6.185	4.40E-05	
	*Rdh16*	Retinol dehydrogenase 16 (all-trans)	NM_199208	6.886	1.48E-05	
	*Rdh7*	Retinol dehydrogenase 7	NM_133543	5.287	3.08E-04	
	*Ros1*	ROS proto-oncogene 1, receptor tyrosine kinase	NM_012874	−10.407	4.25E-05	
	*Smpd3*	Sphingomyelin phosphodiesterase 3, neutral membrane	NM_053605	7.269	5.09E-04	
	*Spib*	Spi-B transcription factor (Spi-1/PU.1 related)	NM_001024286	−4.252	3.10E-04	(12,43)
	*Tekt3*	Tektin 3	NM_001024739	4.718	9.84E-03	
	*Tp73*	Tumor protein p73	NM_001108696	7.167	9.02E-03	(14,37,45)
	*Tspan1*	Tetraspanin 1	NM_001004236	−5.135	1.29E-07	(37)
	*Zbtb16*	Zinc finger and BTB domain containing 16	NM_001013181	12.123	2.98E-03	(9,10,24)
	*Zfp597*	Zinc finger protein 597	NM_153732	6.367	2.49E-06	
G-3	*Agt*	Angiotensinogen (serpin peptidase inhibitor, clade A, member 8)	NM_134432	4.423	3.71E-04	(16–18, 25,39)
	*Agtrap*	Angiotensin II receptor-associated protein	NM_001007654	−37.832	2.17E-05	(16,17)
	*Apoh*	Apolipoprotein H (β-2-glycoprotein I)	NM_001009626	51.420	2.89E-04	(19,26)
	*Cyp1a1*	Cytochrome P450, family 1, subfamily a, polypeptide 1	NM_012540	6.820	6.72E-04	
	*Ephx2*	Epoxide hydrolase 2, cytoplasmic	NM_022936	−12.769	2.99E-05	(27)
	*Hrg*	Histidine-rich glycoprotein	NM_133428	4.578	2.22E-05	(28)
	*Kng2*	Kininogen 2	NM_012741	4.436	4.58E-04	
	*Ryr1*	Ryanodine receptor 1 (skeletal)	XM_008759293	-4.021	1.77E-03	(29)
	*Sds*	Serine dehydratase	NM_053962	6.414	5.51E-06	
	*Vegfb*	Vascular endothelial growth factor B	NM_053549	273.620	2.85E-06	(30)
	*Zfp597*	Zinc finger protein 597	NM_153732	5.870	1.51E-04	
G-4	*Agtrap*	Angiotensin II receptor-associated protein	NM_001007654	30.146	5.83E-07	(16,17)
	*Apoh*	Apolipoprotein H (β-2-glycoprotein I)	NM_001009626	−69.883	7.70E-04	(19)
	*Banp*	Btg3 associated nuclear protein	NM_001106191	−5.011	9.76E-06	(31,51)
	*Ccr1*	Chemokine (C-C motif) receptor 1	NM_020542	−4.607	6.91E-03	
	*Ephx2*	Epoxide hydrolase 2, cytoplasmic	NM_022936	13.574	4.56E-06	(31)
	*Gabrr1*	Gamma-aminobutyric acid (GABA) A receptor, rho 1	NM_017291	4.618	9.48E-03	
	*Hrg*	Histidine-rich glycoprotein	NM_133428	−5.193	4.95E-05	
	*Lilrb3l*	Leukocyte immunoglobulin-like receptor, subfamily B (with TM and ITIM domains), member 3-like	NM_001037357	6.280	3.48E-04	
	*Micb*	MHC class I polypeptide-related sequence B	NM_001017468	5.071	2.74E-03	
	*Nfe2l3*	Nuclear factor, erythroid 2-like 3	XM_231763	−4.386	1.95E-04	
	*Rbfox1*	RNA binding protein, fox 1 homolog (*C. elegans*) 1	NM_001106974	4.016	8.02E-04	(32)
	*Rxrg*	Retinoid X receptor gamma	NM_031765	−23.771	2.66E-06	(31,33)
	*Ryr1*	Ryanodine receptor 1 (skeletal)	XM_008759293	6.837	1.84E-04	(31)
	*Vegfb*	Vascular endothelial growth factor B	NM_053549	−263.762	4.74E-07	(52)
	*Zbtb16*	Zinc finger and BTB domain containing 16	NM_001013181	−6.754	3.92E-04	(32,39)
	*Zfp597*	Zinc finger protein 597	NM_153732	−6.243	3.99E-06	

SHRs, spontaneously hypertensive rats; SHRSP, stroke-prone SHRs; GS, gene symbol; FC, fold change of >4- or <−4-fold in expression.

## Abbreviations

ADHD: attention-deficit hyperactivity disorder
BP: blood pressure
DAVID: Database for Annotation, Visualization and Integrated Discovery
FC: fold change
GEO: Gene Expression Omnibus
GO: Gene Ontology
GS: gene symbol
IPA: Ingenuity Pathway Analysis
RT-qPCR: reverse transcription-quantitative polymerase chain reaction
SHRs: spontaneously hypertensive rats
SHRSP: stroke-prone SHRs
WKY rats: (normotensive) Wistar-Kyoto rats
